# Supplementary material for: Extracellular Vesicle Membrane Protein Profiling and Targeted Mass Spectrometry Unveil CD59 and Tetraspanin 9 as Novel Plasma Biomarkers for Detection of Colorectal Cancer
Source: Cancers (Basel). 2022 Dec 28;15(1):177. doi: 10.3390/cancers15010177 (PMC9818822; doi:10.3390/cancers15010177)
Supplement: Supplementary file 1 [file cancers-15-00177-s001.zip › Supplementary figures.pdf]

Fig. S1

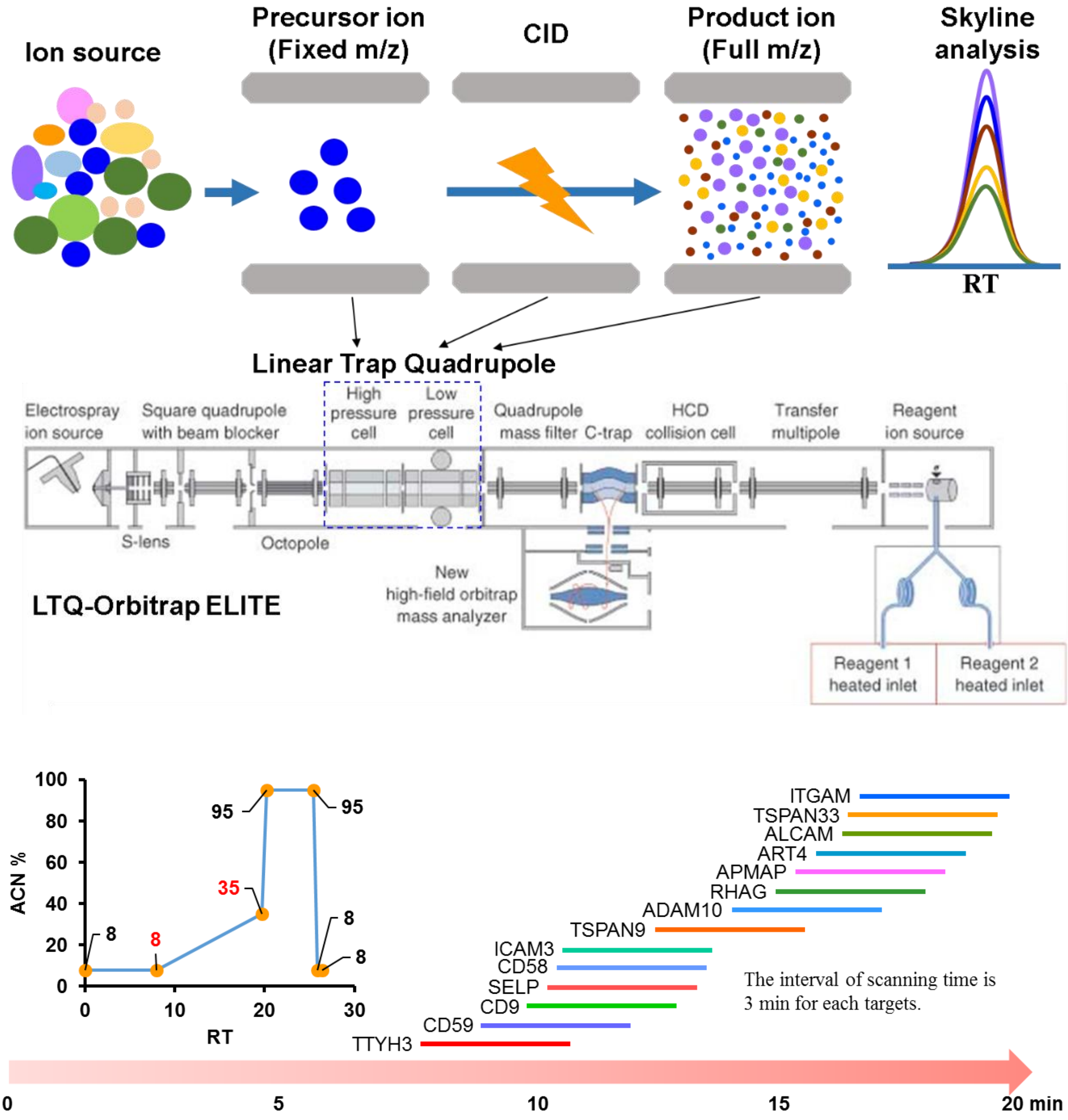

Figure S1. Schematic representation of the product ion scanning (PIS) assay used to quantify 13 target peptides and CD9 in plasma EV samples.

Fig. S2

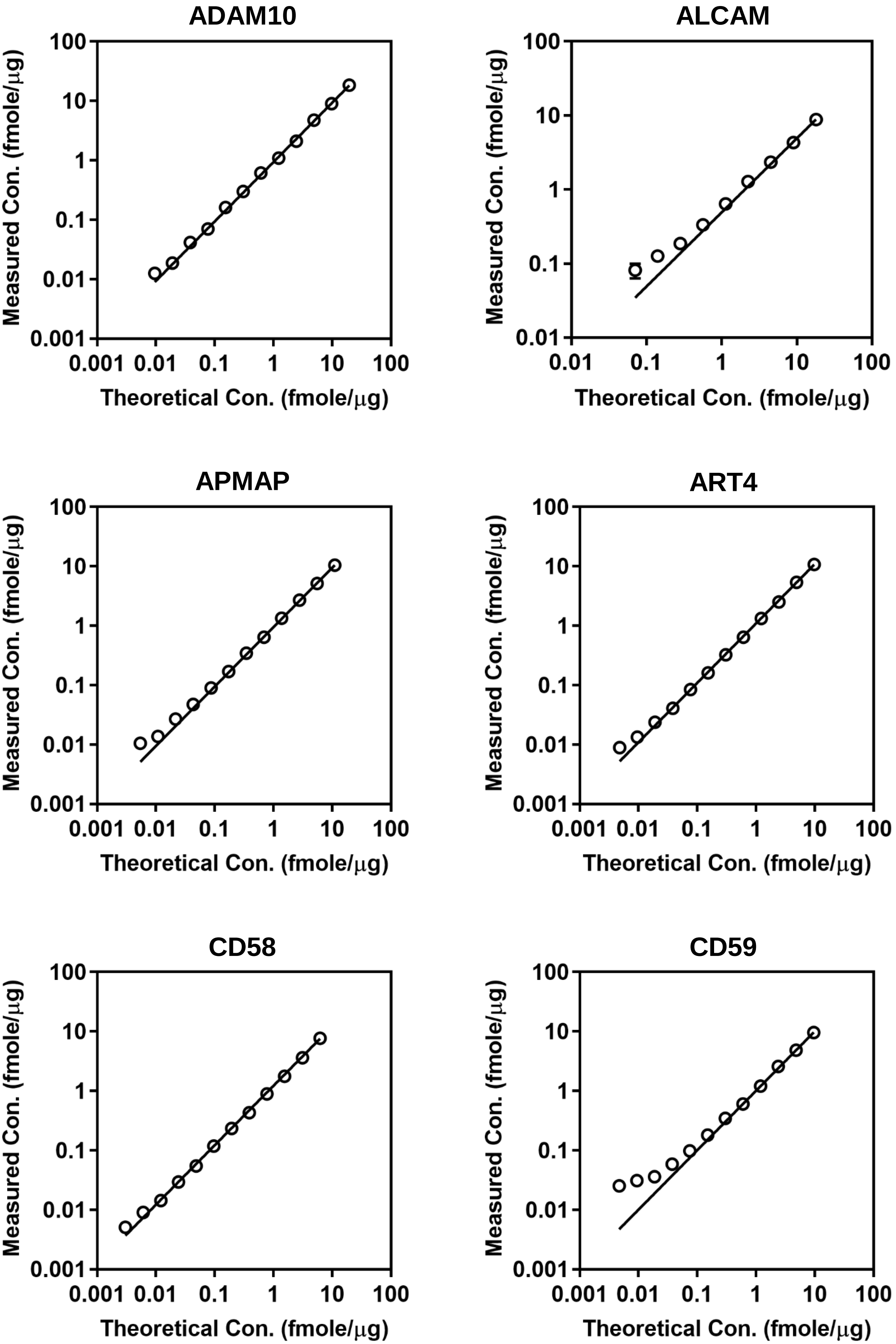
ADAM10
ALCAM
APMAP
ART4
CD58
CD59
Measured Con. (fmole/μg)
Theoretical Con. (fmole/μg)
0.001
0.01
0.1
1
10
100

Fig. S2 (continued)

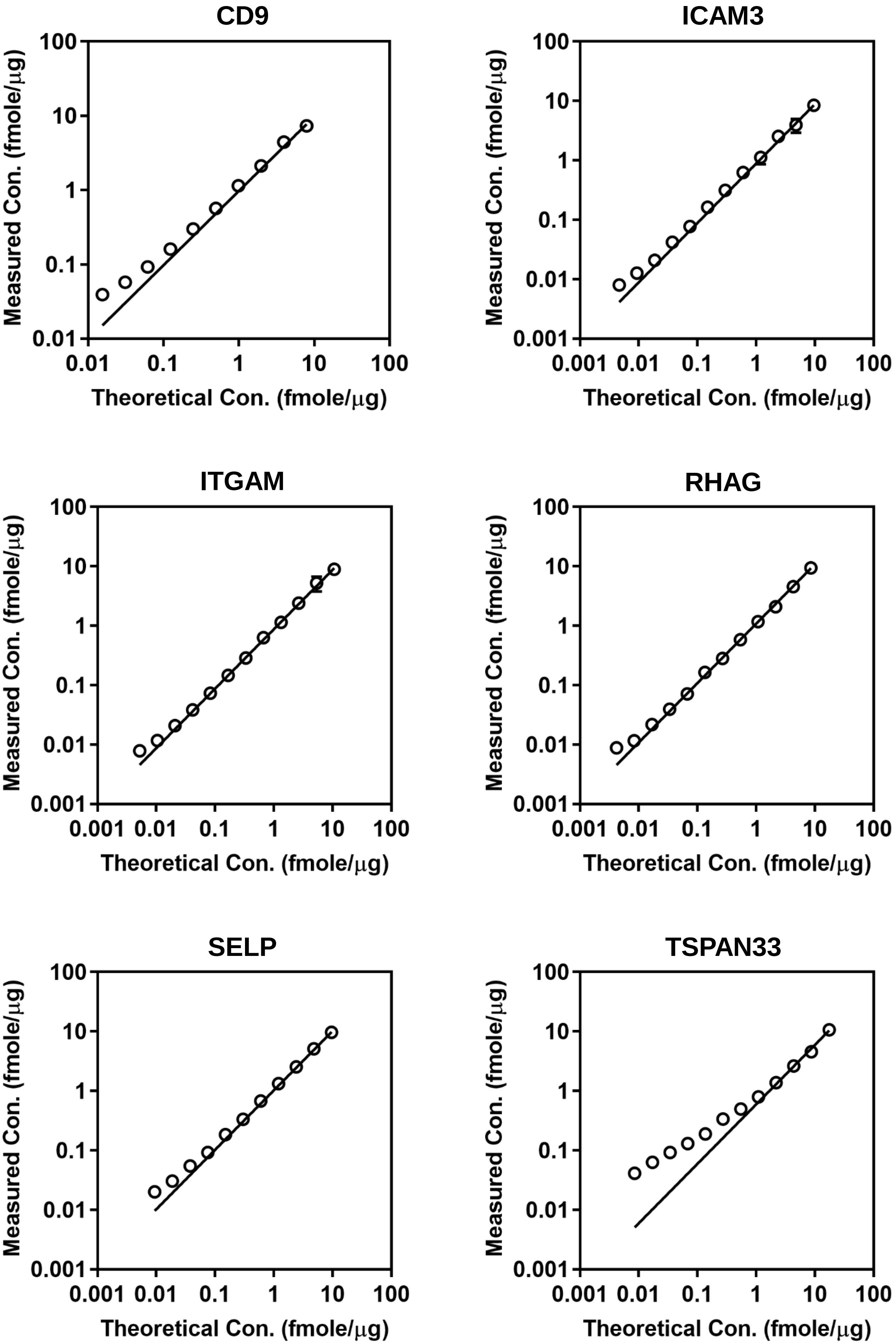

Fig. S2 (continued)

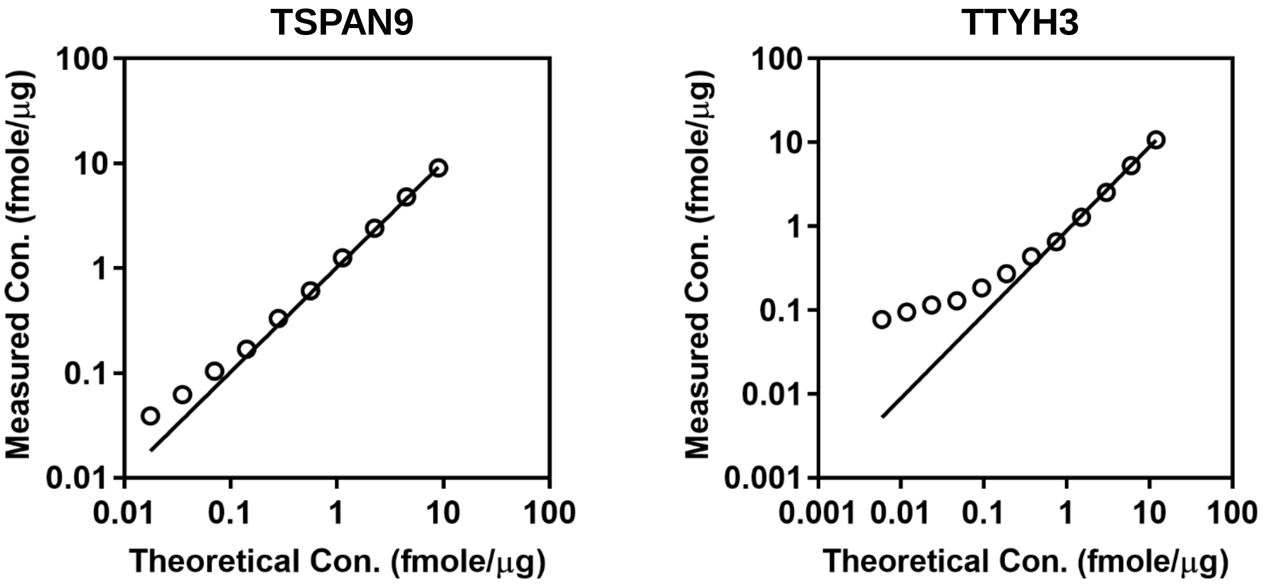

Figure S2. Response curves of the PIS-MS assay for 14 targets in an EV background matrix harvested from HC plasma.

Fourteen heavy peptide mixtures were serially diluted (0, 0.049, 0.098, 0.195, 0.391, 0.781, 1.563, 3.125, 6.25, 12.5, 25, 50 and 100 fmol), and then each dilution point was suspended in a digest of EV protein (1 μg) and mixed with 10 fmol of 14-plex light peptide mixtures. Each dilution point was prepared in quintuplicate and analyzed by LC-PIS-MS. Plot diagrams are displayed as heavy/light peak-area ratios (measured concentration on the y-axis) for summarizing peak areas of 10 transitions (obtained using Skyline) of individual peptides as a function of the theoretical concentration (x-axis).

Fig. S3

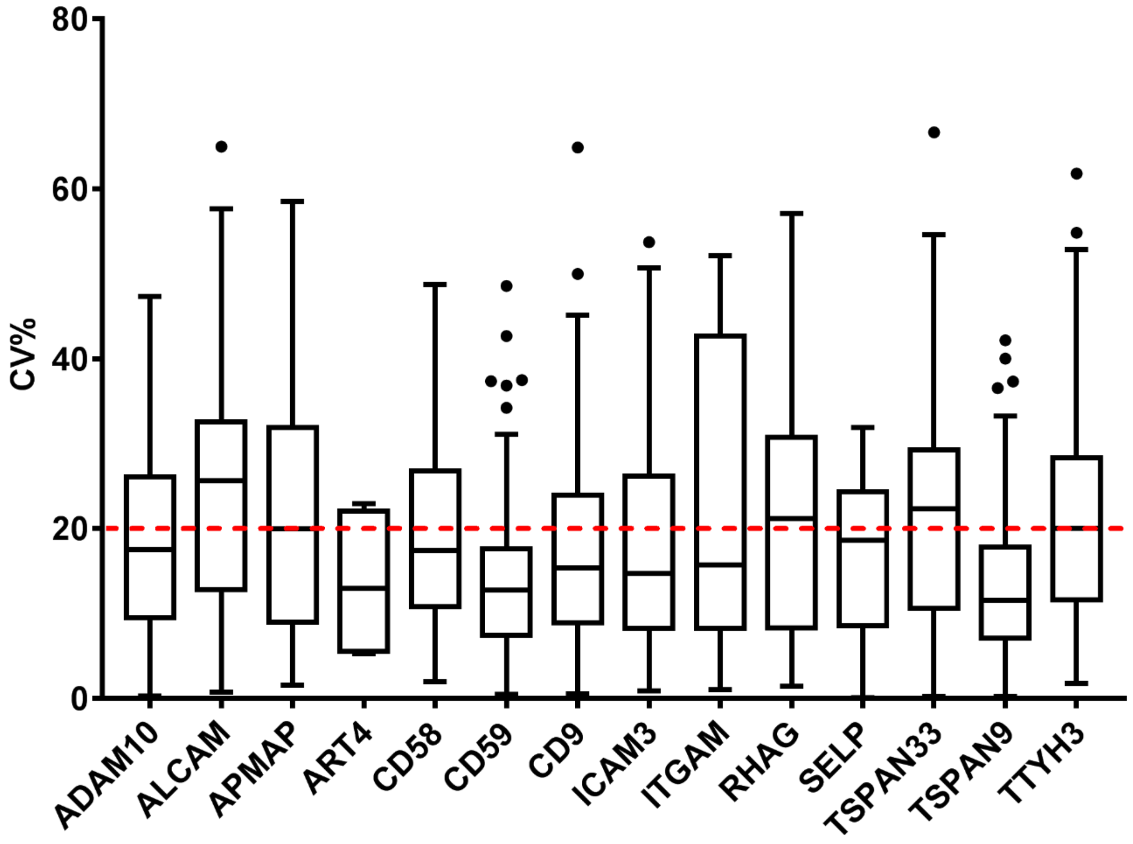

Figure S3. Precision of the 14-plex PIS assay in 153 plasma samples. Red dotted line indicates a CV of 20%.

Fig. S4

(A)

Protein	LLOQ	HC	CRC
ADAM10	0.702	31(38.8%)	68(93.2%)
ALCAM	4.893	2(2.5%)	4(5.5%)
APMAP	0.439	21(26.3%)	40(54.8%)
ART4	0.295	0(0%)	11(15.1%)
CD58	0.192	17(21.3%)	48(65.8%)
CD59	0.386	48(60%)	73(100%)
CD9	1.415	36(45%)	46(63%)
ICAM3	0.518	41(51.3%)	22(30.1%)
ITGAM	1.196	1(1.3%)	19(26%)
RHAG	0.499	8(10%)	8(11%)
SELP	2.142	13(16.3%)	15(20.5%)
TSPAN33	0.294	37(46.3%)	23(31.5%)
TSPAN9	1.110	18(22.5%)	64(87.7%)
TTYH3	1.673	27(33.8%)	22(30.1%)

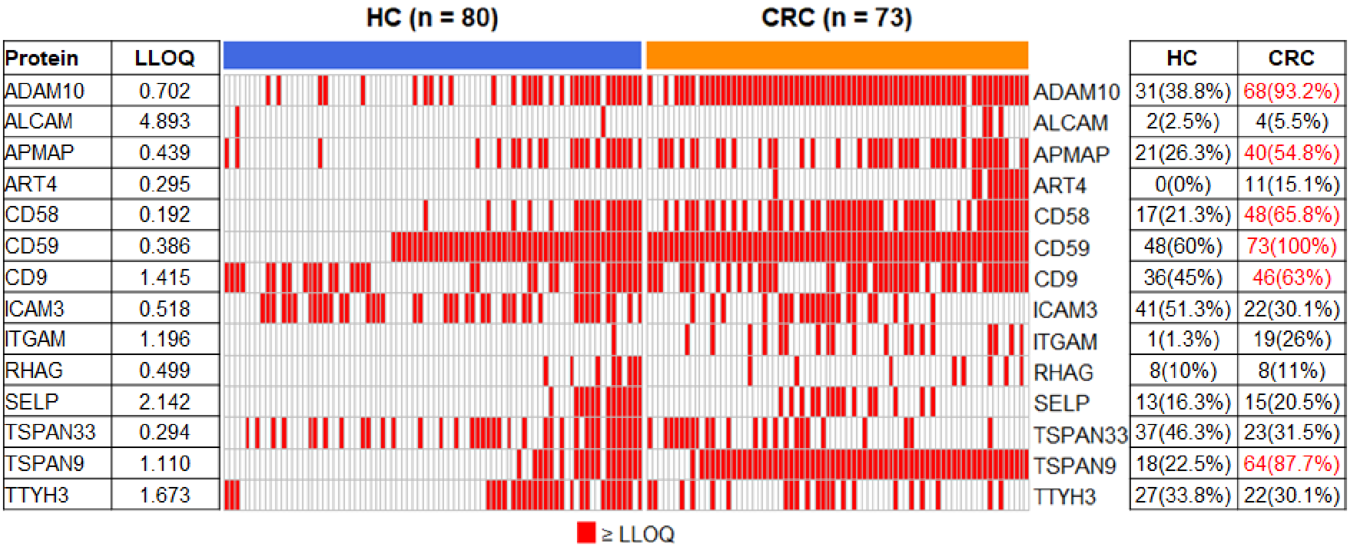

(B)

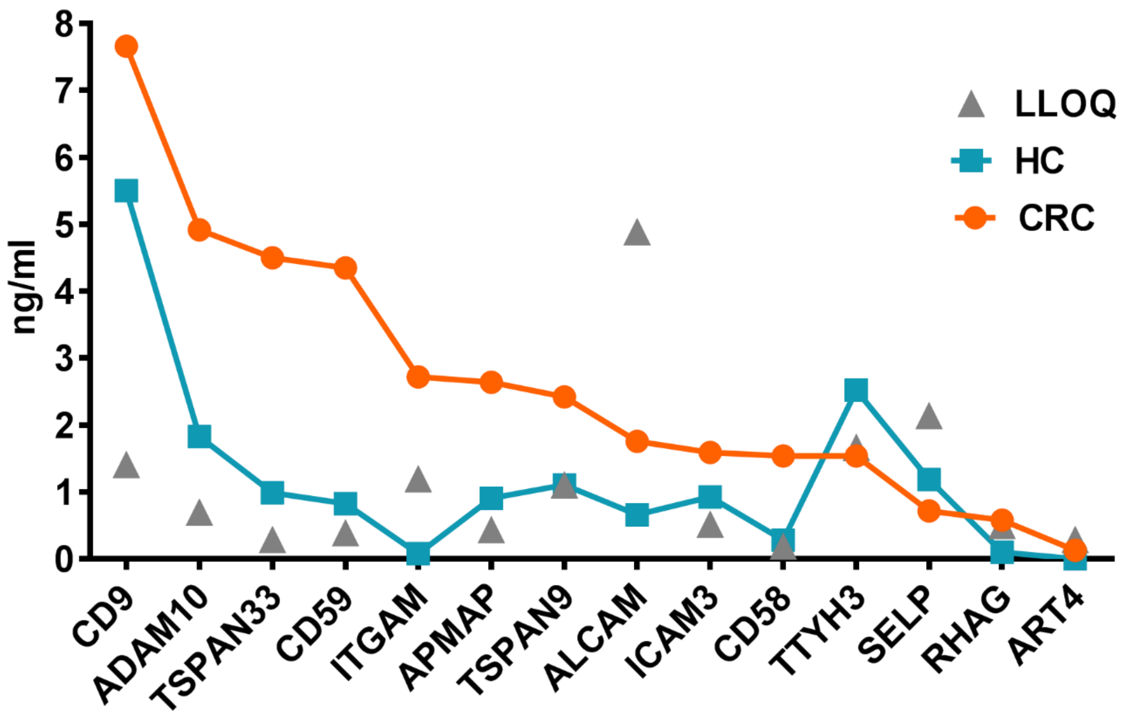

Figure S4. Analysis of the 14 target proteins in plasma EV samples from 80 HCs and 73 CRC patients. (A) An overview of target protein concentration distributions across the two groups. Cases with indicated protein concentration > LLOQ are denoted in red. (B) Comparison of the average concentrations of the 14 target proteins in plasma EV samples from HCs and CRC patients with their LLOQ values.
