# Supplementary material for: Extracellular Vesicle Membrane Protein Profiling and Targeted Mass Spectrometry Unveil CD59 and Tetraspanin 9 as Novel Plasma Biomarkers for Detection of Colorectal Cancer
Source: Cancers (Basel). 2022 Dec 28;15(1):177. doi: 10.3390/cancers15010177 (PMC9818822; doi:10.3390/cancers15010177)
Supplement: Supplementary file 1 [file cancers-15-00177-s001.zip › Table S1.pdf]

Table S1. Clinicopathological characteristics of the subjects from whom plasma samples were obtained and used in this study

		For discovery experiment (n=60)		For verification experiment (n=153)	
Characteristics		HC	CRC	HC	CRC
Gender	Female	13	10	36	37
	Male	17	20	44	36
Age (years)[a]	-	57.43 ± 10.76	60.40 ± 10.70	58.51 ± 7.79	57.77 ± 4.69
Tumor stage (T)	T1	-	2	-	14
	T2	-	3	-	9
	T3	-	18	-	42
	T4	-	7	-	8
Lymph node metastasis (N)	No	-	13	-	37
	Yes	-	17	-	36
Distant metastasis (M)	No	-	23	-	57
	Yes	-	7	-	16
TNM stage	I	-	3	-	18
	II	-	9	-	14
	III	-	11	-	25
	IV	-	7	-	16

[a] Data are shown in mean ± standard deviation (SD).
