# Supplementary figures and images for: Extracellular Vesicle Membrane Protein Profiling and Targeted Mass Spectrometry Unveil CD59 and Tetraspanin 9 as Novel Plasma Biomarkers for Detection of Colorectal Cancer
Source: Cancers (Basel). 2022 Dec 28;15(1):177. doi: 10.3390/cancers15010177 (PMC9818822; doi:10.3390/cancers15010177)

The original images corresponding to the Figure 2E in the main article

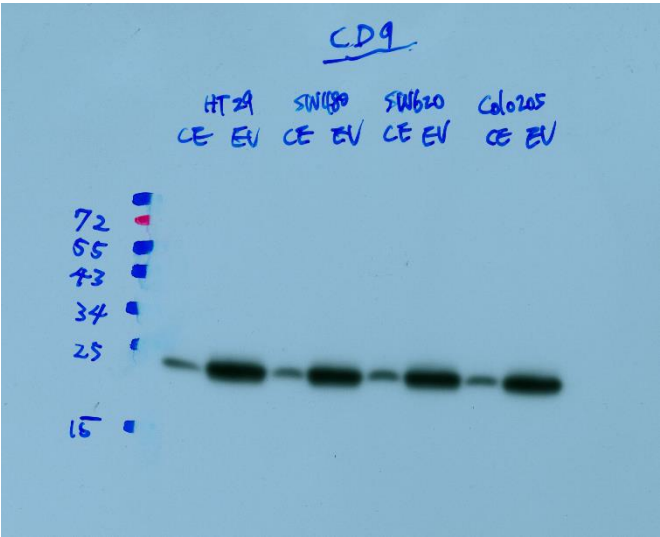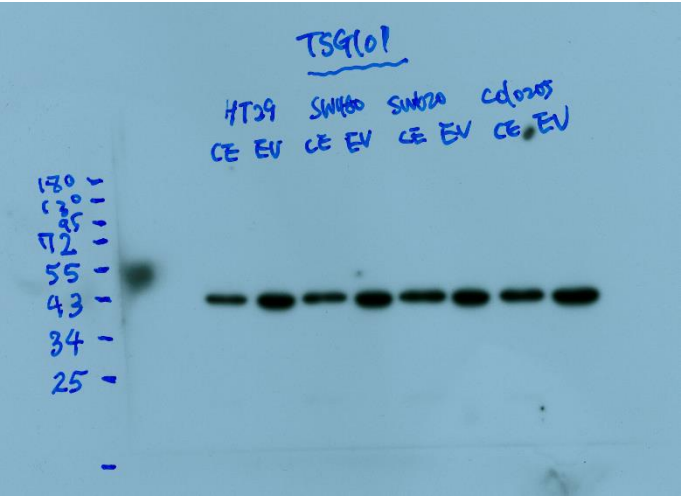

Supplement: Supplementary file 1 [file cancers-15-00177-s001.zip › File S1 Original blots.pdf]
